# Histone deacetylase HDA-4-mediated epigenetic regulation in space-flown *C. elegans*

**DOI:** 10.1038/s41526-021-00163-7

**Published:** 2021-09-01

**Authors:** Atsushi Higashitani, Toko Hashizume, Mai Takiura, Nahoko Higashitani, Mika Teranishi, Rika Oshima, Sachiko Yano, Kana Kuriyama, Akira Higashibata

**Affiliations:** 1 Graduate school of Life Sciences, Tohoku University, Sendai, Miyagi Japan; 2 Advanced Engineering Services Co. Ltd., Tsukuba, Ibaraki Japan; 3 Human Spaceflight Technology Directorate, Japan Aerospace Exploration Agency, Tsukuba, Ibaraki Japan; 4 Japan Space Forum, Shin-ochanomizu Urban Trinity Building 2F, Tokyo, Japan

**Keywords:** Molecular biology, Chromatin

## Abstract

Epigenetic changes during long-term spaceflight are beginning to be studied by NASA’s twin astronauts and other model organisms. Here, we evaluate the epigenetic regulation of gene expression in space-flown *C. elegans* by comparing wild type and histone deacetylase (*hda*)*-4* mutants. Expression levels of 39 genes were consistently upregulated in all four generations of adult *hda-4* mutants grown under microgravity compared with artificial Earth-like gravity (1*G*). In contrast, in the wild type, microgravity-induced upregulation of these genes occurred a little. Among these genes, 11 contain the domain of unknown function 19 (DUF-19) and are located in a cluster on chromosome V. When compared with the 1*G* condition, histone H3 trimethylation at lysine 27 (H3K27me3) increased under microgravity in the DUF-19 containing genes *T20D4.12* to *4.10* locus in wild-type adults. On the other hand, this increase was also observed in the *hda-4* mutant, but the level was significantly reduced. The body length of wild-type adults decreased slightly but significantly when grown under microgravity. This decrease was even more pronounced with the *hda-4* mutant. In ground-based experiments, one of the *T20D4.11* overexpressing strains significantly reduced body length and also caused larval growth retardation and arrest. These results indicate that under microgravity, *C. elegans* activates histone deacetylase HDA-4 to suppress overregulation of several genes, including the DUF-19 family. In other words, the expression of certain genes, including negative regulators of growth and development, is epigenetically fine-tuned to adapt to the space microgravity.

## Introduction

Environmental factors can influence not only genome instability but also epigenetic mechanisms, such as DNA methylation and histone modifications, leading to altered gene expression, certain diseases, and aging^1–5^. Microgravity-induced transcriptional alterations have been observed in several spaceflight experiments, suggesting that epigenetic changes might occur in the space environment^6–8^. However, research on epigenetic regulation of plants and metazoans in the space environment has only just begun to be reported^9,10^.

Recent multi-omics analyses from more than 50 astronauts and hundreds of spaceflight samples show that mitochondrial dysregulation is a consistent and central hub of the biological effects of spaceflight^11^. In 2009, our *C. elegans* RNA Interference Space Experiment (CERISE) was conducted to evaluate RNAi activity and physiological changes during development from L1 larvae to adult under microgravity or 1*G* conditions^12^. Both transcriptome and proteome analyses showed that the expression of muscle proteins, cytoskeletal elements, and mitochondrial metabolic enzymes were decreased by microgravity^12^. Intriguingly, mitochondrial perturbations have been reported to activate epigenetic modifying enzymes such as histone H3K27 histone demethylases JMJD-3.1 and JMHJD-1.2 in *C. elegans*^13^. In addition, mammalian obesity and diabetic subjects have been reported to alter DNA methylation levels of several gene promoters, PGC1α and Tfam (the regulators of mitochondrial biogenesis)^14,15^. One of the intriguing results from the NASA Twin study shows that certain epigenetic changes in DNA methylation of immune and oxidative stress-related pathways occurred during the astronaut’s second 6-month mission^10^.

DNA methylation at the fifth position of cytosine (5mC) plays an important role as an epigenetic mark in mammals, but it is thought to be at low levels or absent in the fruit fly *Drosophila melanogaster* and the nematode *Caenorhabditis elegans*^16,17^. In contrast, histone modifications are widely conserved in eukaryotes, including yeasts, plants, and metazoans^18–22^. Histone modifications can be associated with transcriptional activation or repression through chromatin structural dynamics. For instance, acetylation and trimethylation of histone H3 at lysine 27 (H3K27ac and H3K27me3) regulate transcriptional activation and repression, respectively^23,24^. The level of the H3K27me3 modification is controlled by cooperation with histone deacetylases (HDACs) and methyltransferases (HMT)^25,26^. In particular, class IIa HDACs 4 and 5 in rat skeletal muscles are activated by hindlimb suspension, and it leads to decreased expression of myosin heavy chain and increased expression of MuRF-1 and MAFbox E3-ligases, which are involved in rat muscular atrophy^27,28^.

Here we conducted spaceflight experiments in the International Space Station (ISS): we grew and analyzed four consecutive generations of synchronous culture of wild-type and histone deacetylase (*hda*)*-4* mutant *C. elegans*. In wild-type *C. elegans*, HDA-4 is expressed in the body-wall muscles and several neurons, including motor neurons and other somatic nervous systems; it is an ortholog of mammalian HDAC4 and 5^29–32^. In our previous spaceflight experiment, CERISE, the expression of *hda-4* in wild type was slightly but significantly increased under microgravity (1.49 times that observed under the artificial 1*G*)^12^. However, this CERISE space experiment investigated the effect of microgravity on gene and protein expression only in the first generation, which grows from L1 larvae to adults. It was unclear whether HDA-4 dependent epigenetic changes in response to the microgravity environment would occur not only in the first generation but also across generations. Here, we compared the wild-type and the *hda-4 (ok518)* mutant transcriptomes over four generations in two test environments under microgravity and artificial 1*G* conditions. Our results clarified the existence of HDAC-mediated epigenetic regulation in *C. elegans* that is reproducible in all generations under microgravity conditions.

## Results

### Changes in gene expression between wild type and hda-4 mutant under microgravity conditions

**Fig. 1:**
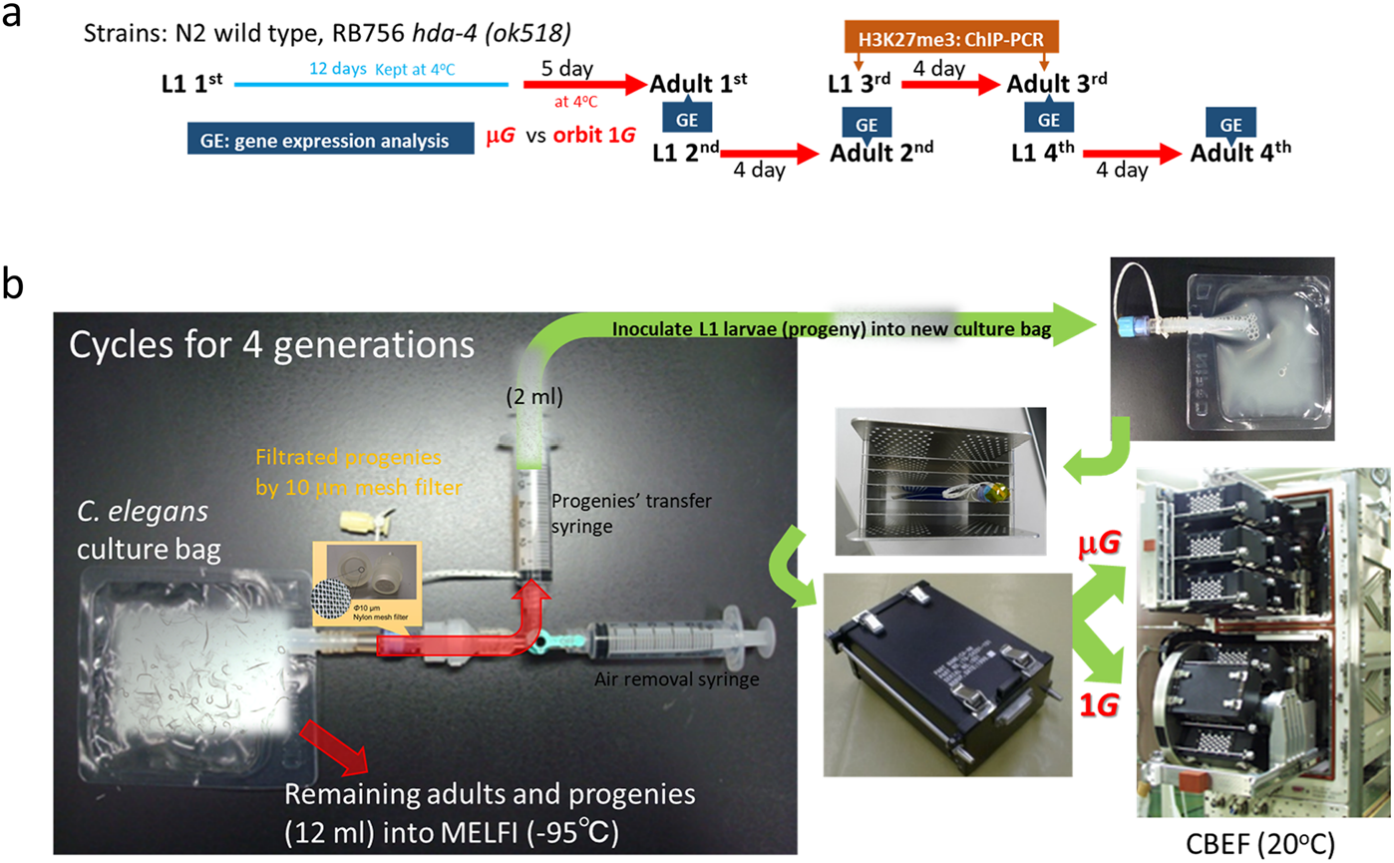
Schematic diagram and illustration of the spaceflight experiment to assess microgravity impact on epigenetic regulation in *C. elegans*. **a** Diagram shows the timeline of the spaceflight experiment “EPIGENETICS”. **b** The start of the experiment was initiated by injecting the L1 larva solution in a syringe into a culture bag and incubating in an upper microgravity box and an artificial 1*G* centrifuge box of CBEF (Cell Biology Experiment Facility). After culturing for 5 days (1st generation) and 4 days (2nd to 4th generations), 2 ml of L1 larval progenies were aspirated with a syringe while being filtered through a 10 μm mesh filter, and transferred to a new culture bag. The remaining culture bags (adults and L1 progenies mixture) were frozen at −95 °C in MELFI (Minus Eighty degree Celsius Laboratory Freezer).

Here, we succeeded in synchronously culturing four consecutive generations of *C. elegans* in space microgravity by using a filtration system (Fig. 1). A 10-μm mesh filter sufficiently separated the parental adults and their progenies, and only progenies could be transferred to the new culture bag. The last culture bag contained adults (4th generation) and their progenies (5th generation larvae) (Supplementary Fig. 1).

**Fig. 2:**
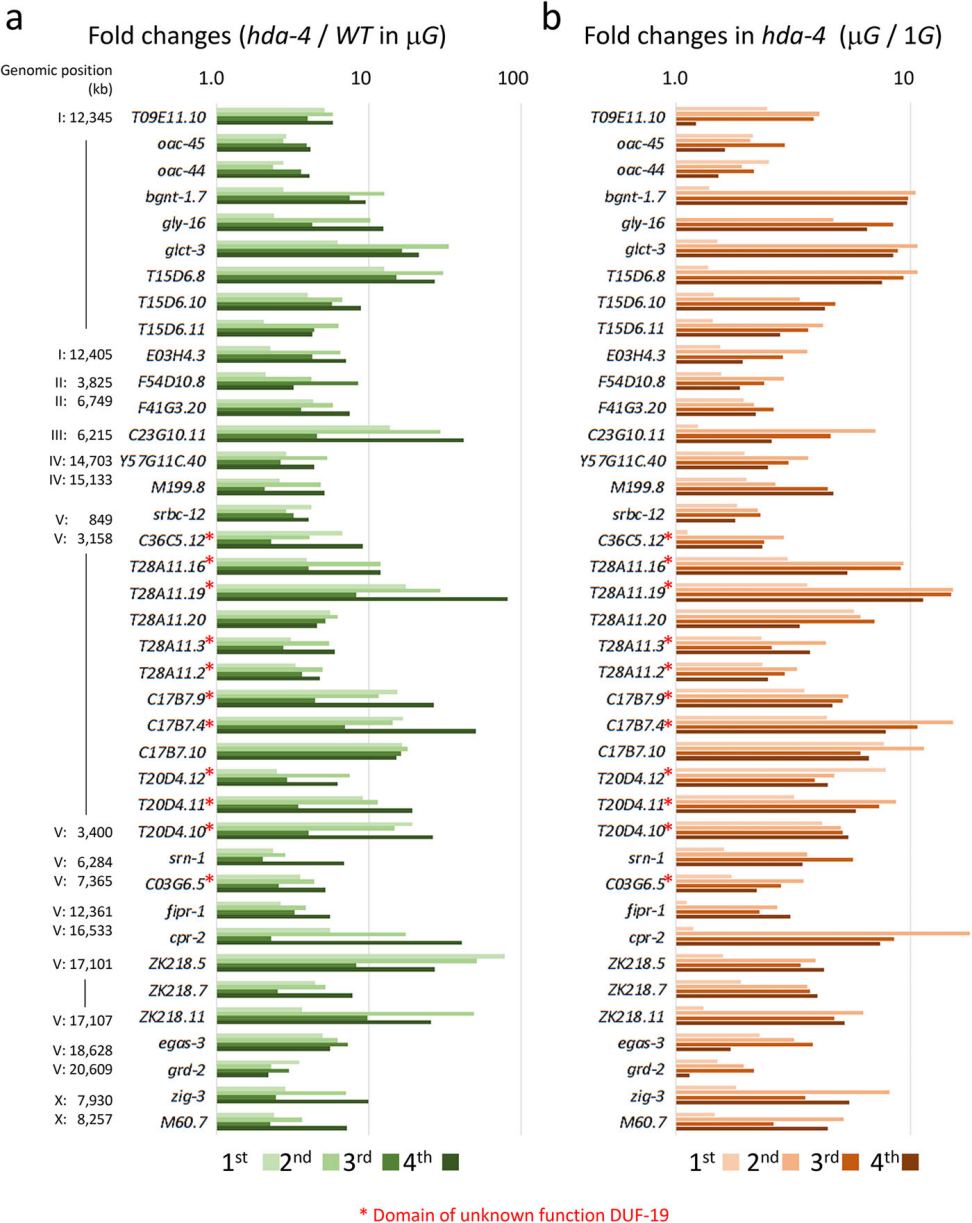
Changes in gene expression induced in *hda-4 (ok518)* mutants grown under microgravity conditions. **a** DNA microarray analysis was compared through four generations of wild-type and *hda-4* mutant strains grown under microgravity in two independent replicate experiments. All 39 genes were significantly upregulated in the *hda-4* mutant (>2.5-fold, *p* < 0.05 using Student’s *t* test). Statistical analysis was performed between the mean values of the four generations of wild-type and *hda-4* mutants. Their chromosome positions and expression levels (fold-change values) were indicated. **b** Changes in gene expression (fold-change values) of 39 genes in the (**a**) of *hda-4* mutants grown under microgravity and artificial 1*G* conditions. Asterisk (*) indicated genes containing a domain of unknown function DUF-19.

DNA microarray analyses were performed to determine the gene expression profiles of wild-type and *hda-4 (ok518)* deletion mutant specimens of adult hermaphrodite *C. elegans* synchronously cultured for four generations under microgravity and artificial 1*G* conditions (Fig. 1). Under microgravity, 39 genes were consistently upregulated (fold-change >2.5; *P* < 0.05, Student’s *t* test) in all four generations of the *hda-4* mutant compared with the equivalent generations of wild type (Fig. 2a). In the mutant, these genes were also consistently upregulated in most (>2/4) generations under microgravity compared with artificial 1*G* (Fig. 2b). These 39 genes were located across all autosomal and sex chromosomes, and some were adjacent cluster genes. This suggests that the expression of these genes was epigenetically suppressed at the adjacent chromosomal level by the action of HDA-4 in response to microgravity. Many of these genes have unknown functions but some share the same domain: e.g., a cluster of 11 genes with the DUF-19 (domain of unknown function 19) on chromosome V (Fig. 2).

**Fig. 3:**
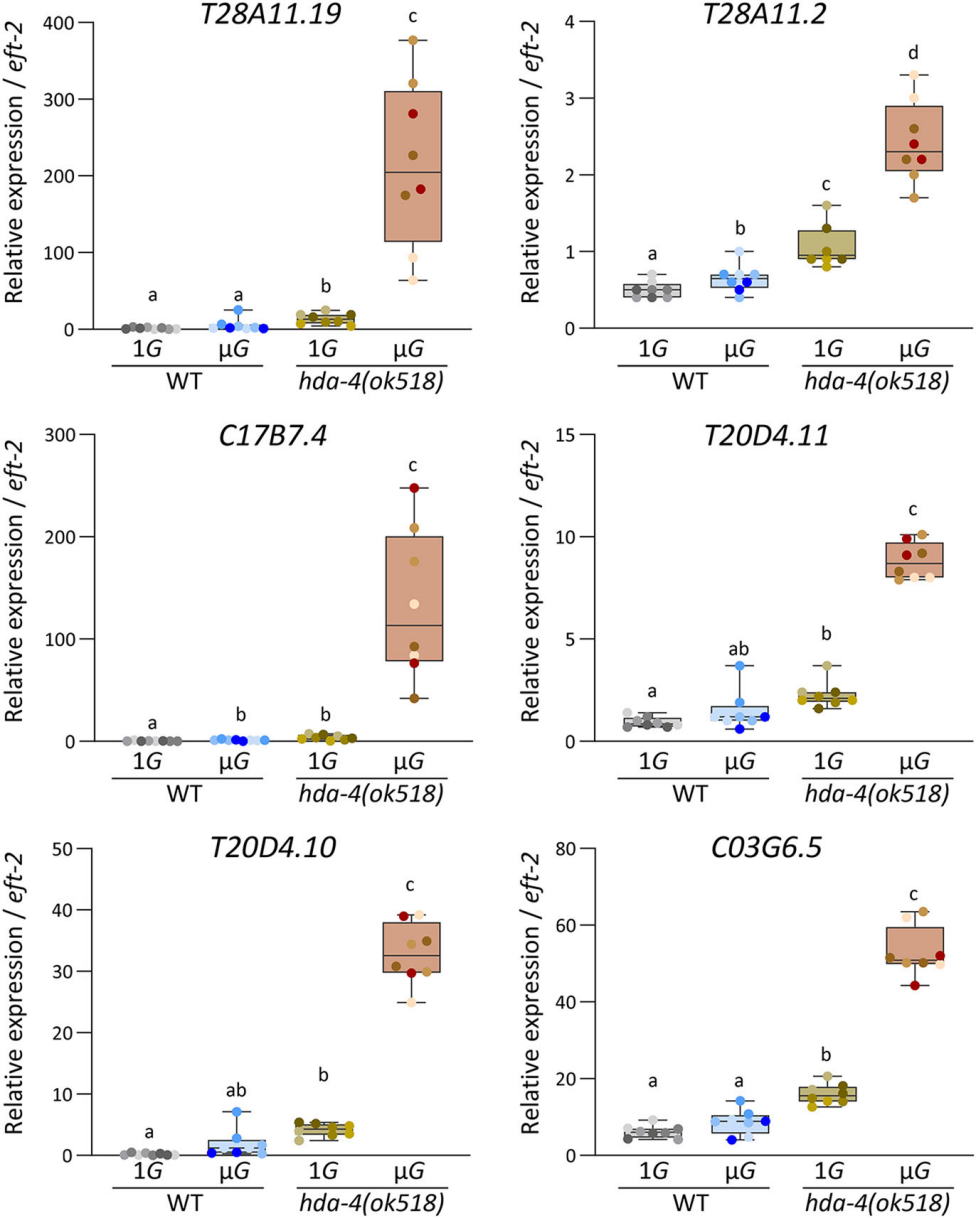
Changes in gene expression of 6 DUF-19-containing genes, *T28A11.19, T28A11.2, C17B7.4, T20D4.11, T20D4.10, and C03G6.5* assessed by quantitative real-time PCR. Expression levels were analyzed in 8 samples each of wild-type and *hda-4* mutants grown in either microgravity or artificial 1*G* (2 independent lines over four generations). The *eft-2* gene was used as the internal standard. Data are shown as box and whiskers to indicate median and range. Statistical analysis was performed in each condition using one-way ANOVA followed by Tukey post hoc test.

To confirm the expression changes, the transcript levels of 6 of the DUF-19–containing genes, *T28A11.19, T28A11.2, C17B7.4, T20D4.11, T20D4.10*, and *C03G6.5*, were examined by real-time reverse transcription polymerase chain reaction (RT-PCR) using two independent space experiment samples. Consistent with the microarray data (Fig. 2), all showed significantly higher expression in the *hda-4 (ok518)* mutant than in the wild type under microgravity, and significantly higher expression in the *hda-4 (ok518)* mutant under microgravity than in the mutant under artificial 1*G* (Fig. 3). Intriguingly, under microgravity conditions, some of the genes in wild-type (*C17B7.4, T28A11.2*) were slightly but significantly upregulated under microgravity compared with artificial 1*G* (Fig. 3).

### Histone H3K27 trimethylation changes in response to microgravity

**Fig. 4:**
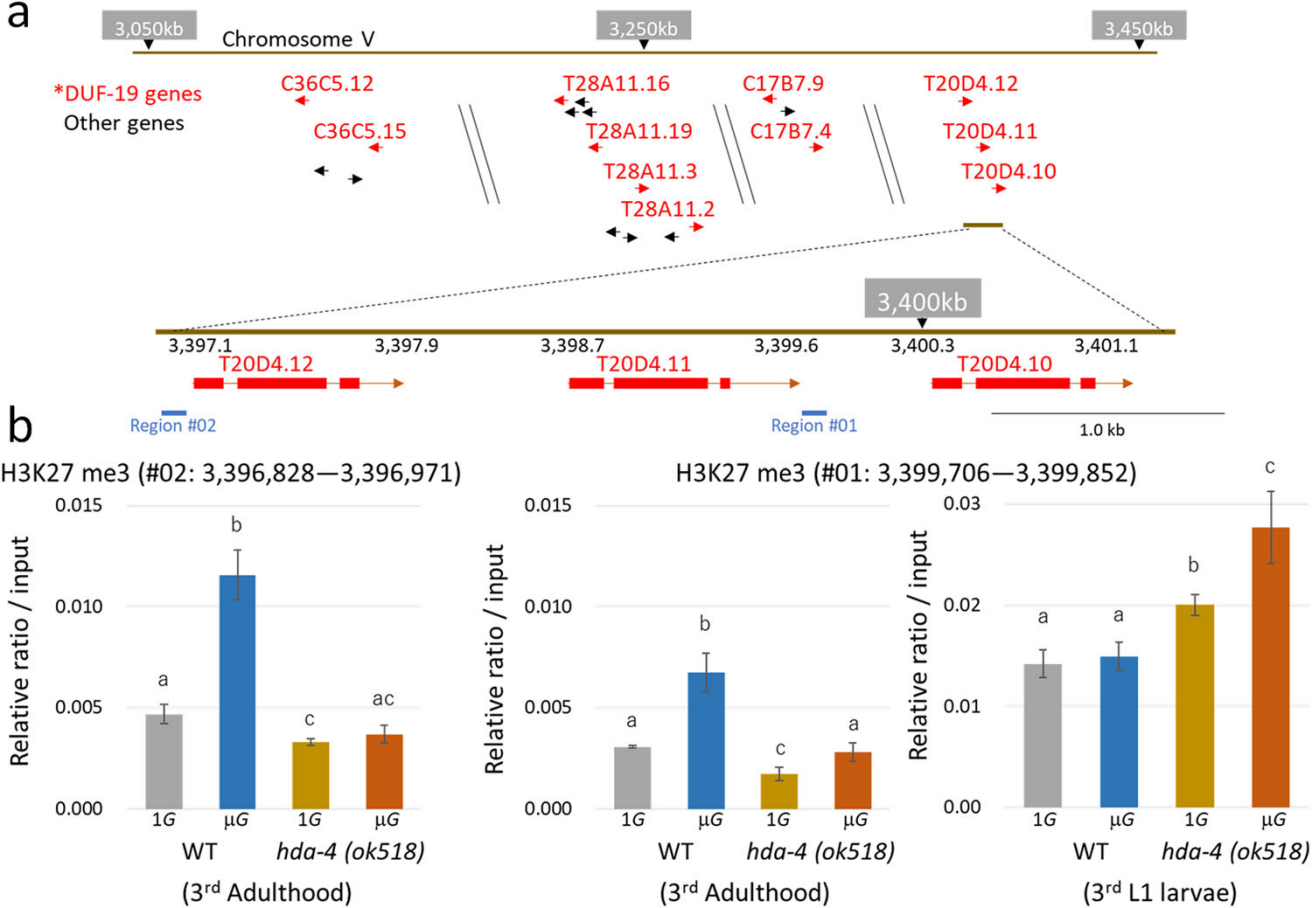
ChIP analysis of H3K27me3 levels at the *T40D4.12-4.10* locus. **a** H3K27me3 levels were analyzed in the intergenic region of the *T40D4.12-4.10* locus on Chromosome V. The red-colored gene is a gene containing the DUF-19 domain. **b** The graphs show the recovery rates for region #01 and #02, as a relative ratio of DNA after ChIP with the anti-H3K27me3 monoclonal antibody compared to the input DNA in adults wild-type and *hda-4* mutant, respectively. Due to the sample limitations, L1 larvae study was limited to analysis of the H3K27me3 status at the region #01. These samples (about 300 worms each) grew as third generation under artificial 1*G* or microgravity conditions. Biological triplications in each sample condition (*n* = 3) were analyzed by ChIP-qPCR using one-way ANOVA followed by Tukey post hoc test. Error bars indicate S.D.

To study changes in H3K27me3 in adult *C. elegans* grown under microgravity, chromatin immunoprecipitation (ChIP)—genomic PCR with anti-H3K27me3 antibody was applied to the intergenic regions of the *T20D4.12* to *4.10* gene locus; two regions, #01 and #02, were studied (Fig. 4a). Specimens analyzed were all 3rd-generation L1 larvae or adults. In wild-type adults, H3K27me3 levels in both regions were significantly higher under microgravity than under artificial 1*G* (Fig. 4b). In the *hda-4* mutant, the basal level of H3K27me3 under artificial 1*G* was lower than that in wild type, and the degree of H3K27me3 induction by microgravity was severely reduced (Fig. 4b). In L1 larvae, H3K27me3 levels in the *hda-4* mutant were higher than in the adult stage, and were slightly but significantly higher than in the wild type under both microgravity and artificial 1*G* (Fig. 4b). These results suggest that the sites examined in the locus of *T20D4.12* to *4.10* are highly tri-methylated at the early larval stage and are maintained under silencing conditions. The state of H3K27me3 then changes via epigenetic enzymes such as HDA-4, if the specimen is subjected to microgravity during growth. We also tested epigenome changes in H3K27ac, H3K27me1, and H3K27m2 in the same locus, but all modification levels were lower and could not be further evaluated due to sample quantitative limitations.

### hda-4 mutant displays pronounced growth suppression in spaceflight and overexpression of the T20D4.11 gene

**Fig. 5:**
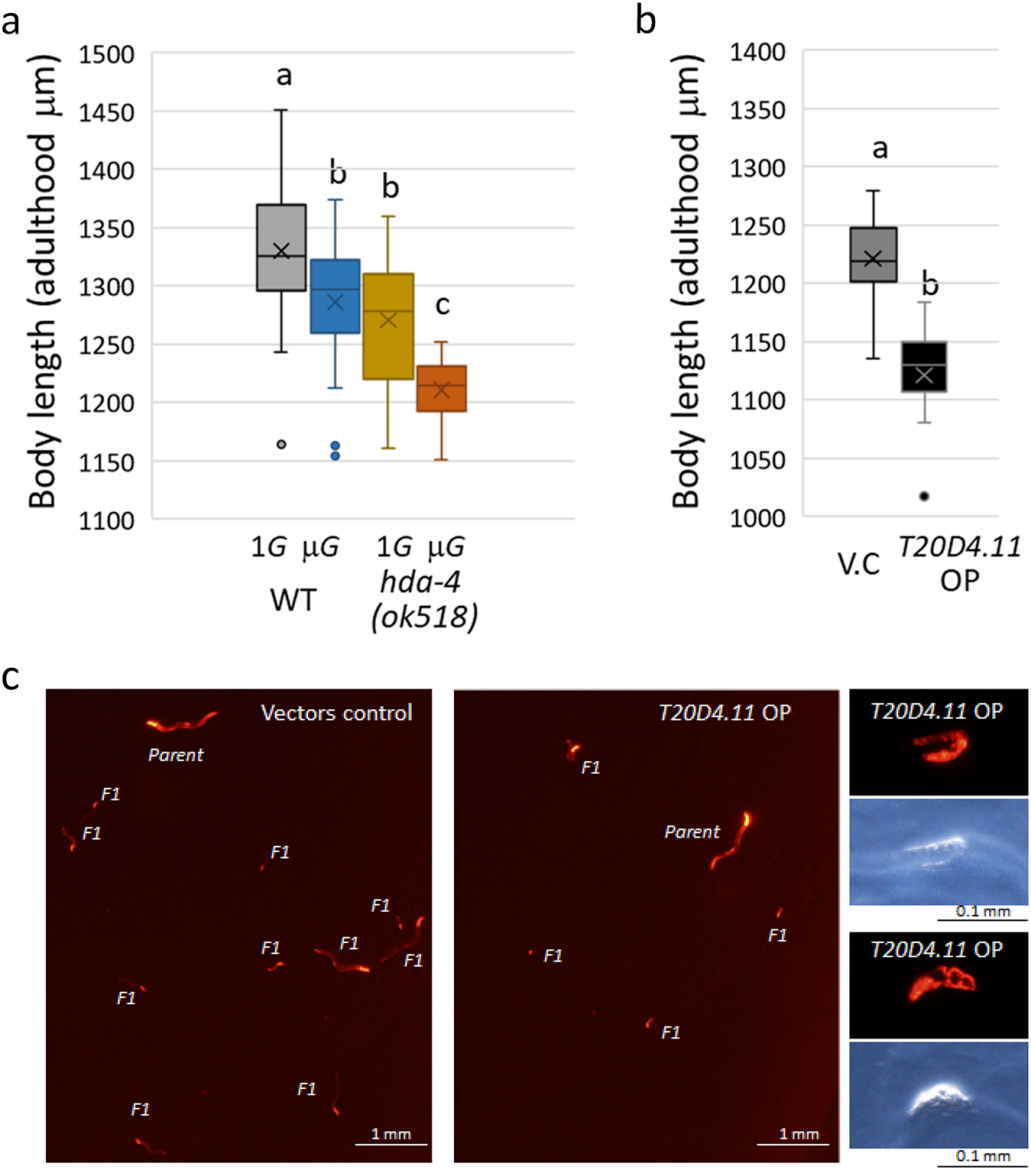
Changes in body length of space-flown *C. elegans*. **a** We plotted the length of 3rd-generation adults of wild-type and *hda-4* mutants grown under artificial 1*G* or microgravity was indicated (*n* = 20<, respectively). Data are shown as box and whiskers to indicate median and range. Statistical analysis was performed using one-way ANOVA followed by Tukey post hoc test. **b** Adult length of transgenic lines of vector control and *T20D4.11* driven by the *myo-3* promoter grown on NGM *E. coli* OP-50 agar plate for 4 days (*n* = 10, *P* < 0.01 using Student’s *t* test). Data are shown as box and whiskers to indicate median and range. **c** Five L1 larvae of vector control and *T20D4.11* transgenic lines were cultured on 6 cm NGM *E. coli* OP-50 agar plates for 7 days. The L1 larva became an adult (Parent) and its progenies (F1) were visualized with injection markers (*Prab-3::mCherry, Pmyo-2::mCherry* and *Pmyo-3::mCherry*, Supplementary Fig. 2). Several sizes of F1 progenies were observed on the vector-control line. On the other hand, in the *T20D4.11* transgenic line, F1 progenies became smaller, and some dead larvae and embryos were observed (right panels).

We have previously discovered that the *C. elegans* body length changes in response to environmental conditions such as fluid dynamics and microgravity^12,33^. Reproducible in this study, the length of wild-type *C. elegans* was slightly but significantly (by 3.3%, *p* = 0.011) reduced in adulthood when they were grown under microgravity compared with artificial 1*G* (Fig. 5a). This reduction was even more pronounced in the *hda-4 (ok518)* mutant (by 4.8%, *p* = 0.00001). In addition, the body thickness of the mutant was significantly reduced (Supplementary Fig. 1).

There are more than 80 paralogs of DUF-19–containing genes in the *C. elegans* genome. Despite comprehensive RNAi experiments involving each of these genes, no knockdown-related phenotype has been observed^34^. This is because there are many DUF-19 gene families in the genome, so even if each gene function is disrupted, the phenotype may not be obtained. In addition, it may be difficult to observe the knockdown phenotype if the original genetic function adversely affects growth and survival and is usually rarely expressed. Therefore, we investigated the phenomena caused by overproduction of the DUF-19–containing gene *T20D4.11* driven by the promoter of the body-wall muscle myosin *myo-3* gene (Supplementary Fig. 2). The body length in adults was significantly lower (by 8.2%, *p* = 0.0001) in one of the *T20D4.11* overexpression lines compared with the vector control (Fig. 5b). Therefore, we consider that the reason that the *hda-4* mutant is shorter than the wild-type grown at microgravity is probably the higher expression of DUF-19-containing genes such as *T20D4.11* (Fig. 3). In addition, the overexpressed adult hermaphrodite reduced brood size, the progenies did not hatch frequently (embryonic or early larval lethality), and many survived showed growth retardation (Fig. 5c). These results suggest that DUF-19-containing genes function as negative regulators of growth and development in response to some stress and environmental conditions.

## Discussion

Here we carried out a spaceflight experiment using *C. elegans* to study whether some of the transcriptional changes caused by microgravity are controlled by epigenetic changes. Comprehensive gene expression analyses using the *hda-4* genetic mutant and wild-type revealed that there are 39 genes whose expression in adult *C. elegans* in response to space microgravity is suppressed by the action of HDA-4.

Polycomb Repression Complex 2 (PRC2), which includes histone lysine methyltransferase (HMT/KMT), induces trimethylation of histone H3 lysine 27 and recruits HDAC and DNA methyltransferase (DNMT) to further inhibit transcription^35–37^. In addition, HMT/KMT promotes trimethylation after deacetylation of lysine residues of histone by HDAC^38^. Therefore, HDACs and HMT/KMTs work together to cause epigenetic silencing. Consistent with this, a significant increase in H3K27me3 at the *T20D4.12* - *T20D4.10* locus under microgravity was observed in wild-type adults compared with artificial 1*G*, but a little in the *hda-4* mutant (Fig. 4). We consider that the induction levels of these genes in microgravity-grown *C. elegans* are downregulated by HDA-4 to adjust to appropriate expression levels in the space environment. This microgravity-induced epigenetic modification will be reset in the germ cells of the next generation. In *C. elegans* spermatogenesis, epigenetic modifications occur throughout the genome^39^, and PRC2 maintains the state of H3K27me3 during embryogenesis^40^. In the embryo, H3K27me3 is enriched across genes that were silent in the parental germline^41^. Nine HDACs have been identified in the *C. elegans* genome, of which HDA-1 is primarily involved in germ cell development and embryogenesis^42^. HDA-1 is essential, unlike HDA-4, and knockdown-related phenotypes have been observed in embryonic lethal and defects in gonadogenesis^34^. Therefore, the increase in H3K27me3 at the *T20D4.12* to *T20D4.10* gene locus in L1 larvae may be due to HDA-1-mediated epigenome modification. In addition, this methylation may occur more strongly in adult germ cells with reduced H3K27me3 levels in the absence of HDA-4. Due to the sample limitations, this study was limited to analysis of the H3K27me3 status of 3rd-generation L1 larvae and adults, but future space experiments with *C. elegans* will examine more global epigenetic changes in response to microgravity.

Merkwirth et al.^13^ reported that perturbations in the mitochondrial electron transport chain enhance the expression of the H3K27 histone demethylases JMJD-3.1 and JMHJD-1.2 in *C. elegans*. This converts the H3K27me3 inactive chromosomes into the H3K27me1 active chromosomes, which transcriptionally induces several effectors of unfolding protein response in the mitochondria. Interestingly, at least 19 genes among the 39 genes identified in this study are reported to be induced by JMJD-3.1 under mitochondrial perturbation^13^. In particular, the DUF-19-containing genes *C03G6.5*, *C17B7.4*, *C36C5.12*, *T20D4.10*, *T20D.4.11*, *T20D4.12*, *T28A11.2*, *T28A11.16*, *T28A11.19*, and *T28A11.20* are more than sevenfold upregulated by JMJD-3.1. Since here we found that overexpression of one of these genes, *T20D4.11*, negatively affected growth and development, we propose that a series of DUF-19-containing genes may be involved in growth inhibition under mitochondrial stress conditions.

**Fig. 6:**
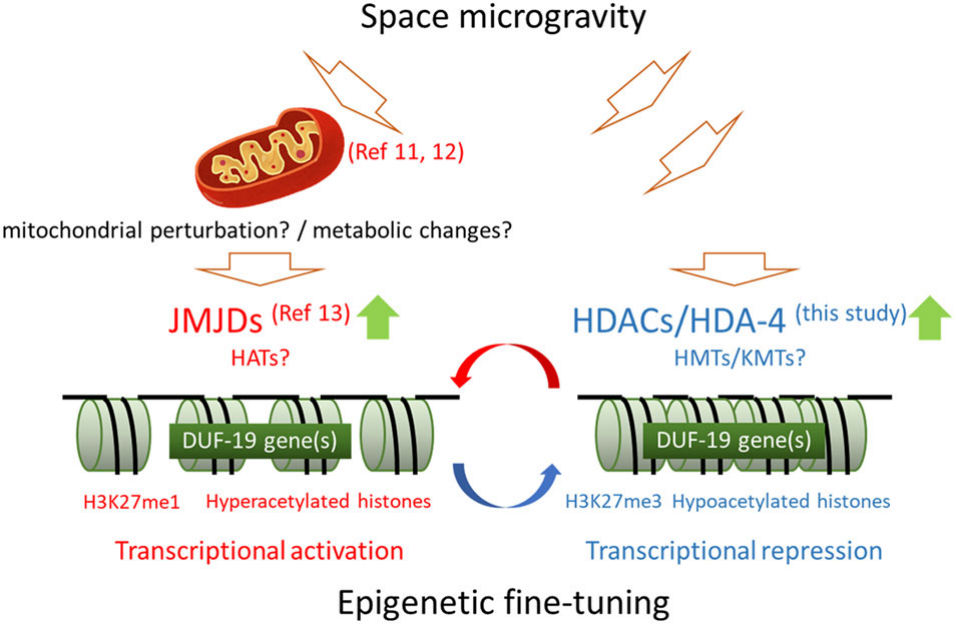
Schematic illustration of epigenetic control by HDACs in space microgravity. The expression of certain genes, including negative regulators of growth and development such as the DUF-19 gene(s), is epigenetically fine-tuned to adapt to the space microgravity in *C. elegans*.

We have previously reported that space-flown *C. elegans* display reduced levels of mitochondrial enzymes as well as muscular and cytoskeletal proteins^12^. Therefore, it is speculated that mitochondrial activity is somewhat reduced and epigenetic modification by JMJDs is promoted in all four generations. It leads to upregulation of the expression of several genes, including the genes containing DUF-19. In the wild-type grown under microgravity, histone deacetylase HDA-4 and KMT are assumed to be activated in parallel, and conversely, H3K27me3 epigenetic modification is enhanced and the expression level of these genes is suppressed. Namely, certain genes including DUF-19 family, which are negative regulators of growth and development, are epigenetically fine-tuned via histone modifications to adapt to the microgravity environment (Fig. 6).

In conclusion, here we clarified that HDAC-mediated epigenetics occurs during the growth of a metazoan species in the space microgravity environment. In the future, we expect that comprehensive tissue-specific epigenome changes associated with various histone modifications will be revealed in relation to the pathophysiological effects of long-term spaceflight.

## Materials and methods

### Spaceflight experiment

In this study, the N2 Bristol (wild-type) strain and RB756 *hda-4 (ok518)* deletion mutant were used. To synchronize the growth of the worms, an egg preparation was made in a laboratory at the Kennedy Space Center as described previously^28^. All L1 larvae (each strain, 9,000 worms) were kept in a 1-ml plastic syringe in M9 buffer containing 5 mg/L cholesterol (M9C) until the initiation of the experiment. The synchronized culture was started by injecting 9000 L1 larvae through a NIPRO-manufactured connection port into a NIPRO culture bag containing a 14 ml suspension of *E. coli* OP-50 in S-basal medium (OD_600_ = 5) in the International Space Station (ISS) Japanese Experiment Module KIBO. After culturing at 20 °C for about 4 days in the ISS’s Cell Biology Experiment Facility (CBEF), a 2 ml suspension of the next generation of L1 larvae was filtered through a 10 μm nylon mesh filter (Semitec Corporation, Japan) and inoculated into a new culture bag. The remaining culture bag was frozen at −95 °C in the ISS freezer MELFI. Each culture was repeated 3 times in the same way to produce samples for a total of four generations. Two independent replicate experiments were performed for each strain in microgravity and artificial 1*G* conditions with centrifuge in the CBEF.

### Expression analyses

Approximately 1000 adult worms were collected by thawing the frozen culture samples on ice and washing them three times by natural sedimentation in ice-cold M9 buffer to remove larvae and bacterial food. RNA extraction, microarray analysis, and real-time RT-PCR analysis were performed as described previously^28,29^. The following primer pairs were used for PCR amplification: *T28A11.19* (forward: 5ʹ-CGA GTG AGT TAG AGA GAA GAA GAA TTA GAA-3ʹ, reverse: 5ʹ-TCT GCT CCA AAT CCA GAA AAA GT-3ʹ), *T28A11.2* (forward: 5ʹ-AAA AGA CAA CTG CAT GAA AAA GGA-3ʹ, reverse: 5ʹ-GCC GAC TCC GAT GAA ATG A-3ʹ), *C17B7.4* (forward: 5ʹ-CGA ATT GTG CAA AAA CAT GGT T-3ʹ, reverse: 5ʹ-GCG AGT CCT CCT CTC CAC AAG-3ʹ), *T20D4.11* (forward: 5ʹ-AGA ACT TGT GGA GGA GCC ATGT-3ʹ, reverse: 5ʹ-AGT CAGC GTC GCA TTG TTT G-3ʹ), *T20D4.10* (forward: 5ʹ-CAG TTG CGA CTG GAG CAG AA-3ʹ, reverse: 5ʹ-GGA CAG ACC CAT GAT GCA AGA-3ʹ), *C03G6.5* (forward: 5ʹ-GCATGAAGAAGGAGATTACTGAAACA-3ʹ, reverse: 5ʹ-ACC GGA AAG ATT GAC AAA TTG C-3ʹ), and *eft-2* (forward: 5ʹ-GAC GCT ATC CAC AGA GGA GG-3ʹ, reverse: 5ʹ-TTC CTG TGA CCT GAG ACT CC-3ʹ). The real-time RT-PCR data were normalized to *eft-2*, which encodes an actin isoform, as a housekeeping gene. The analyses were performed in technical triplicate for each of the eight culture lines (four generations, two independent replicate experiments).

### Body length measurement of space-flown and transgenic adults

The body lengths of wild-type and *hda-4* mutant *C. elegans* that were space-flown, and *C. elegans* that harbored the *T20D4.11* transgene and remained on Earth, were quantified using an Olympus BX51 microscope system with a DP73 digital camera (Olympus, Tokyo, Japan) as described previously^28,29^. Observations were performed immediately after thawing the frozen samples for spaceflight and after fixing the transgenic samples with sodium azide. More than 20 worms were randomly selected for each condition of spaceflight samples.

### ChIP analysis of H3K27me3 levels

ChIP assays were performed using ~300 adults from the 3rd-generation in each experimental condition, monoclonal antibody against H3K27ac, H3K27me1, H3K27m2, and H3K27me3 (MAB Institute Inc. Japan. Cat. No. MABI0309, MABI0321, MABI0324, and MABI0323), and a ChIP solution kit with anti-mouse IgG magnetic beads and control mouse IgG (MAB Institute Inc. Japan. Cat. No. MABI0822). ChIP-qPCR was performed with optimized PCR primer sets at the *T40D4.12 to 4.10* locus: region #01 primer set (forward: 5ʹ-AAC AAT TCC GGC ATC AAC AT-3ʹ, reverse: 5ʹ-TCT GCC AAC TGT CGT TGT TC-3ʹ) and region #02 primer set (forward: 5ʹ-AAA AAG AGT CAC TTG TAA CAG GAA CA-3ʹ, reverse: 5ʹ-CAG TCA CTG GTG CGG TGT AA-3ʹ).

### Statistical analysis

Statistical analyses were performed using one-way ANOVA followed by Tukey post hoc tests in RStudio software and Student’s *t* test. The minimum *p* value for significance was 0.05. Similar alphabet(s) in any two groups indicate no significance and different alphabets in any two groups represent significant difference between the two groups.

### Reporting summary

Further information on research design is available in the Nature Research Reporting Summary linked to this article.

## Supplementary information

Supplementary Information

Reporting Summary

## Data Availability

The data of this study are available from the authors upon reasonable request. All global gene expression data are MIAME (Minimum Information about a Microarray Experiment) compliant and are deposited in the GEO (Gene Expression Omnibus) database as the accession number of GSE173985.
