# Supplementary material for: Histone deacetylase HDA-4-mediated epigenetic regulation in space-flown C. elegans
Source: NPJ Microgravity. 2021 Sep 1;7:33. doi: 10.1038/s41526-021-00163-7 (PMC8410859; doi:10.1038/s41526-021-00163-7)
Supplement: Supplementary file 1 — Supplementary Information [file 41526_2021_163_MOESM1_ESM.pdf]

Supplemental Figures

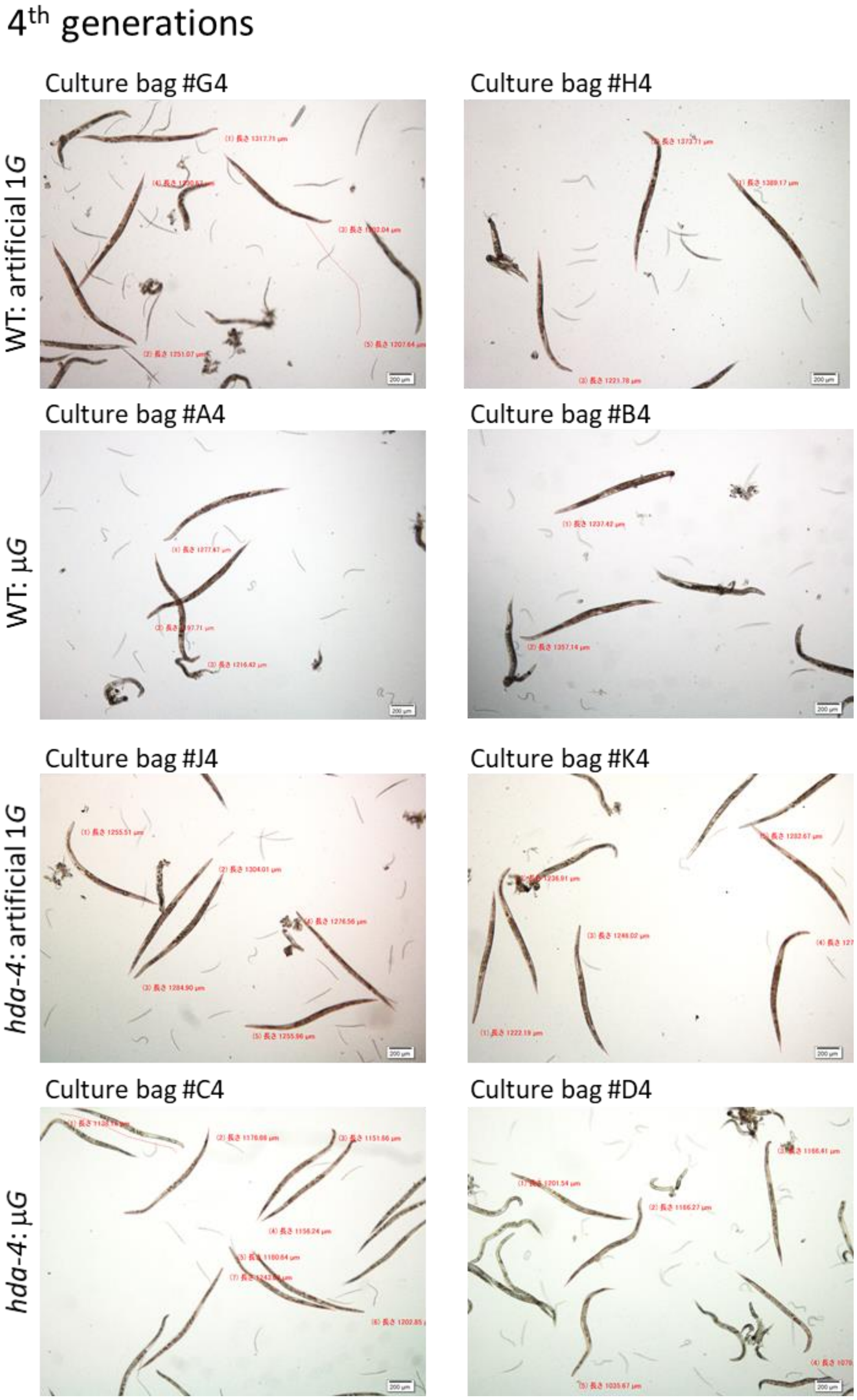

Supplemental Fig. 1 Recovered *C. elegans* space-flown samples, wild type and *hda-4* mutant at 4th generations.

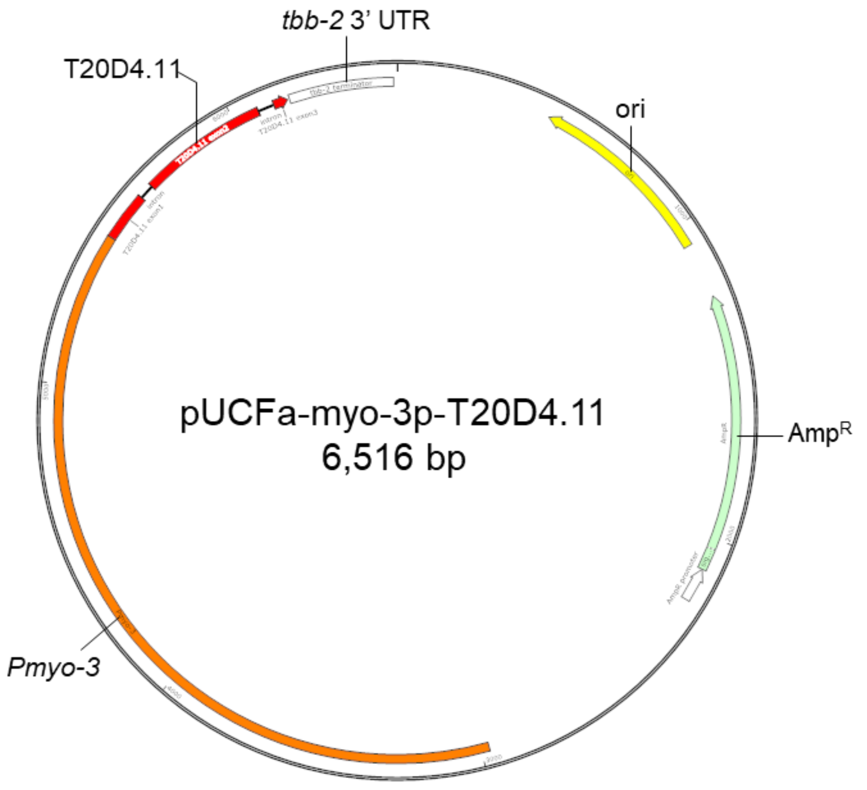

pGH8 (*Prab-3::mCherry::unc-54UTR*)
pCFJ90 (*Pmyo-2::mCherry::unc-54UTR*)
pCFJ104 (*Pmyo-3::mCherry::unc-54UTR*)

Supplemental Fig. 2 A plasmid map of the T20D4.11 transgene regulated by *myo-3* promoter. Other plasmids, pGH8, pCFJ90, and pCFJ104 were also used as markers to screen transgenic lines.
