# Supplementary material for: Histone deacetylase HDA-4-mediated epigenetic regulation in space-flown C. elegans
Source: NPJ Microgravity. 2021 Sep 1;7:33. doi: 10.1038/s41526-021-00163-7 (PMC8410859; doi:10.1038/s41526-021-00163-7)
Supplement: Supplementary file 2 — Reporting Summary [file 41526_2021_163_MOESM2_ESM.pdf]

nature portfolio

Corresponding author(s): Atsushi Higashitani, Akira Higashibata

Last updated by author(s): Jun 30, 2021

Reporting Summary

Nature Portfolio wishes to improve the reproducibility of the work that we publish. This form provides structure for consistency and transparency in reporting. For further information on Nature Portfolio policies, see our Editorial Policies and the Editorial Policy Checklist.

Statistics

For all statistical analyses, confirm that the following items are present in the figure legend, table legend, main text, or Methods section.

n/a	Confirmed	
☐	☒	The exact sample size (*n*) for each experimental group/condition, given as a discrete number and unit of measurement
☐	☒	A statement on whether measurements were taken from distinct samples or whether the same sample was measured repeatedly
☐	☒	The statistical test(s) used AND whether they are one- or two-sided<br>*Only common tests should be described solely by name; describe more complex techniques in the Methods section.*
☐	☒	A description of all covariates tested
☐	☒	A description of any assumptions or corrections, such as tests of normality and adjustment for multiple comparisons
☐	☒	A full description of the statistical parameters including central tendency (e.g. means) or other basic estimates (e.g. regression coefficient) AND variation (e.g. standard deviation) or associated estimates of uncertainty (e.g. confidence intervals)
☐	☒	For null hypothesis testing, the test statistic (e.g. *F*, *t*, *r*) with confidence intervals, effect sizes, degrees of freedom and *P* value noted<br>*Give P values as exact values whenever suitable.*
☒	☐	For Bayesian analysis, information on the choice of priors and Markov chain Monte Carlo settings
☒	☐	For hierarchical and complex designs, identification of the appropriate level for tests and full reporting of outcomes
☒	☐	Estimates of effect sizes (e.g. Cohen's *d*, Pearson's *r*), indicating how they were calculated

Our web collection on statistics for biologists contains articles on many of the points above.

Software and code

Policy information about availability of computer code

Data collection	n/a
Data analysis	RStudio software

For manuscripts utilizing custom algorithms or software that are central to the research but not yet described in published literature, software must be made available to editors and reviewers. We strongly encourage code deposition in a community repository (e.g. GitHub). See the Nature Portfolio guidelines for submitting code & software for further information.

Data

Policy information about availability of data

All manuscripts must include a data availability statement. This statement should provide the following information, where applicable:

- Accession codes, unique identifiers, or web links for publicly available datasets
- A description of any restrictions on data availability
- For clinical datasets or third party data, please ensure that the statement adheres to our policy

The data of this study are available from the authors upon reasonable request. All global gene expression data are MIAME (Minimum Information about a Microarray Experiment) compliant and are deposited in the GEO (Gene Expression Omnibus) database as the accession number of GSE173985.

Field-specific reporting

Please select the one below that is the best fit for your research. If you are not sure, read the appropriate sections before making your selection.

☒ Life sciences ☐ Behavioural & social sciences ☐ Ecological, evolutionary & environmental sciences

For a reference copy of the document with all sections, see nature.com/documents/nr-reporting-summary-flat.pdf

Life sciences study design

All studies must disclose on these points even when the disclosure is negative.

Sample size	In this study, the N2 Bristol (wild-type) strain and RB756 hda-4 (ok518) deletion mutant were used. The start of the experiment was initiated by injecting the L1 larva solution (approximately 9,000) in a syringe into a culture bag and incubating in an upper microgravity box and an artificial 1G centrifuge box of CBEF (Cell Biology Experiment Facility). After culturing for 5 days (1st generation) and 4 days (2nd to 4th generations), 2 ml of L1 larval progenies were aspirated with a syringe while being filtered through a 10 µm mesh filter, and transferred to a new culture bag. The remaining culture bags (adults and L1 progenies mixture) were frozen at -95 °C in MELFI (Minus Eighty degree Celsius Laboratory Freezer). For expression analysis, approximately 1,000 adult worms were used in each condition. More than 20 worms were randomly selected for body length measurement in each conditions of spaceflight samples. ChIP assays were performed using approximately 300 adults in each conditions.
Data exclusions	n/a
Replication	The analyses were performed in technical triplicate for each of the 8 culture lines (4 generations, two independent replicate experiments).
Randomization	n/a
Blinding	n/a

Reporting for specific materials, systems and methods

We require information from authors about some types of materials, experimental systems and methods used in many studies. Here, indicate whether each material, system or method listed is relevant to your study. If you are not sure if a list item applies to your research, read the appropriate section before selecting a response.

Materials & experimental systems

n/a	Involved in the study	
☐	☒	Antibodies
☒	☐	Eukaryotic cell lines
☒	☐	Palaeontology and archaeology
☐	☒	Animals and other organisms
☒	☐	Human research participants
☒	☐	Clinical data
☒	☐	Dual use research of concern

Methods

n/a	Involved in the study	
☒	☐	ChIP-seq
☒	☐	Flow cytometry
☒	☐	MRI-based neuroimaging

Antibodies

Antibodies used	Monoclonal antibody against H3K27ac, H3K27me1, H3K27m2, and H3K27me3 (MAB Institute Inc. Japan. Cat. No. MABI0309, MABI0321, MABI0324, and MABI0323), and a ChIP solution kit with anti-mouse IgG magnetic beads and control mouse IgG (MAB Institute Inc. Japan. Cat. No. MABI0822).
Validation	http://www.monoclo.com/

Animals and other organisms

Policy information about studies involving animals; ARRIVE guidelines recommended for reporting animal research

Laboratory animals	n/a (Uses only the nematode C. elegans)
Wild animals	n/a
Field-collected samples	n/a

Ethics oversight | n/a

Note that full information on the approval of the study protocol must also be provided in the manuscript.
